# γδ T cell frequencies are altered in HIV positive pregnant South African women and are associated with preterm birth

**DOI:** 10.1371/journal.pone.0235162

**Published:** 2020-06-25

**Authors:** Charlene Akoto, Christina Y. S. Chan, Krithi Ravi, Wei Zhang, Manu Vatish, Shane A. Norris, Joris Hemelaar

**Affiliations:** 1 Nuffield Department of Women’s & Reproductive Health, University of Oxford, The Women’s Centre, John Radcliffe Hospital, Oxford, United Kingdom; 2 Department of Paediatrics, South African Medical Research Council Developmental Pathways for Health Research Unit, School of Clinical Medicine, University of the Witwatersrand, Johannesburg, South Africa; CEA, FRANCE

## Abstract

**Background:**

Preterm birth is the leading cause of neonatal and child mortality worldwide. Maternal HIV infection and antiretroviral treatment (ART) increase the rate of preterm birth, but the underlying mechanisms remain unknown, limiting progress in prediction, prevention and treatment. While overall γδ T cell levels remain constant, acute HIV infection is associated with a depletion of the Vδ2 subset and an increase in the Vδ1 subset, which do not return to baseline with ART. γδ T cells have also been implicated in adverse pregnancy outcomes and we therefore investigated the potential association between maternal HIV infection, peripheral γδ T cell frequencies and preterm birth.

**Methods:**

Study participants were HIV positive (n = 47) and HIV negative (n = 45) women enrolled in a prospective pregnancy cohort study at Chris Hani Baragwanath Academic Hospital in Soweto, South Africa. Women were enrolled in early pregnancy and gestational age was accurately determined by first trimester ultrasound scan. Peripheral blood samples were collected in each trimester and peripheral blood mononuclear cells isolated. Frequencies of γδ T cells, Vδ1+ and Vδ2+ γδ T cell subsets, and CCR6 chemokine receptor expression were determined by flow cytometry.

**Results:**

Total γδ T cell levels were similar between HIV positive and HIV negative women throughout pregnancy. However, in each trimester maternal HIV infection was associated with reduced levels of the Vδ2+ subset and increased levels of the Vδ1+ subset, leading to a reversal of the Vδ1/Vδ2 ratio. Timing of ART initiation among HIV positive women did not affect levels of γδ T cells, the Vδ1+ and Vδ2+ subsets, or the Vδ1/Vδ2 ratio. Importantly, preterm birth was associated with lower total γδ T cell levels in early pregnancy and γδ T cell frequencies were lowest in HIV positive women who delivered preterm. Moreover, in the first trimester the proportion of Vδ1+ T cells that were CCR6+ was significantly reduced in HIV+ women and women who delivered preterm, resulting in the lowest proportion of CCR6+ Vδ1 T cells in HIV positive women who delivered preterm.

**Conclusions:**

Our findings suggest that altered γδ T cell frequencies may link maternal HIV infection and preterm birth. γδ T cell frequencies in early pregnancy may serve as predictive biomarkers to identify women at risk of delivering preterm.

## Introduction

Preterm birth (PTB) is a syndrome with multiple aetiologies and the leading cause of neonatal and child mortality globally [1, 2]. While PTB accounts for approximately 18% of child deaths annually, the underlying causative mechanisms remain elusive; hampering the development of much needed tools and therapies for the prediction, prevention and treatment of this complex syndrome [1, 2].

Globally, 37 million people are estimated to be living with HIV/AIDS and an estimated 1.4 million are pregnant women, predominantly living in sub-Saharan Africa [3, 4]. In a systematic review and meta-analysis, our group has shown that HIV positive antiretroviral therapy (ART) naive pregnant women experience higher rates of PTB, low birthweight, small-for-gestational age, and stillbirth compared to HIV negative mothers [5]. The introduction of ART is effective at reducing maternal morbidity and mortality as well as mother-to-child transmission, but the impact of HIV on adverse perinatal outcomes is not reversed, and may even be further exacerbated [6–13].

The hallmark of HIV infection is a sustained depletion of CD4+ T cells and immunological dysfunction resulting in an increase in opportunistic infections, other morbidities and death [14]. In addition to CD4+ T cells, a number of other cell types are impacted by HIV infection. γδ T cells comprise 5–10% of circulating T lymphocytes and in humans two major subsets are categorised according to their expression of Vδ chains, namely Vδ1 and Vδ2. These pair with one of seven functional Vγ chains: Vγ2, Vγ3, Vγ4, Vγ5, Vγ8, Vγ9, or Vγ11, with some combinations being more common than others and showing a tropism for particular tissues [15]. For instance, in the peripheral blood Vγ9Vδ2 T cells constitute the majority of γδ T cells and represent 1–10% of circulating lymphocytes, whereas Vδ1 in combination with various Vγ chains are typically found at epithelial surfaces, including those of the liver and skin, and mucosa of the respiratory, digestive and reproductive tracts [16]. γδ T cells respond to endogenous and environmental stress signals in a manner that is independent of classical MHC antigen presentation, allowing a rapid response to activation. γδ T cells have been implicated in a number of effector functions, including the killing and clearance of infected or transformed cells by engagement of FAS receptors, the release of cytotoxic effector molecules such as perforin, granzymes, granulysin and the human cathelicidin LL-37, the secretion of immunomodulatory cytokines involved in anti-microbial, antifungal and anti-parasitic responses, and the activation of other immune effector cells [17–22]. At epithelial surfaces Vδ1 are the major γδ T cell population which recognise ligands including glycolipids presented by CD1 molecules and stress-inducible MHC class I-related chain (MIC) A and MICB molecules [23–25]. They are able to kill a range of epithelial tumours and are thought to play roles in tissue homeostasis and repair [26–28]. Vγ9Vδ2 are activated by both microbial and mammalian sources of phosphoantigens (phosphorylated non-peptide metabolites of the isoprenoid pathway) and respond to pathogens such as *Mycobacterium tuberculosis* as well as tumours [29–31] but are 1,000-fold less sensitive to activation by phosphoantigens produced by mammalian cells [32–34].

A number of groups have shown that HIV infection does not impact overall peripheral γδ T cell numbers when compared to HIV negative individuals [35–38]. However, HIV infection is associated with lower Vδ2 T cell numbers and loss of function and increases in Vδ1 T cells, resulting in a reversal of the Vδ1/Vδ2 ratio, which occurs prior to declines in CD4+ T cell counts [35–41]. Furthermore, the Vδ1/Vδ2 ratio and γδ T cell function do not return to normal with ART [36, 37]. However, whether these HIV/ART-related changes in γδ T cells are also seen during pregnancy is not known.

Vδ1 and Vδ2 γδ T cells are present within the decidua and produce IL-17, which has been implicated in both healthy and adverse pregnancy outcomes [42–46]. Furthermore, in healthy human pregnancies, peripheral γδ T cell numbers are increased in pregnant and postpartum women and γδ T cells are found in the decidua where they produce IL-10 and TGF-β, suggesting a role in the maintenance of pregnancy by contributing to a favourable cytokine environment [47–51]. For instance, peripheral total γδ T cells, as well as TGF-β producing γδ T cells, are reduced in pregnant women with a history of recurrent pregnancy loss [49]. Therefore, considering the HIV-induced changes to γδ T cell ratios and the association of pregnancy loss with decreases in γδ T cell numbers, we aimed to investigate the potential association between maternal HIV infection, peripheral γδ T cell frequencies, and adverse perinatal outcomes, specifically preterm birth.

## Materials and methods

### Patients

Blood samples were obtained from women enrolled in a prospective pregnancy cohort study at Chris Hani Baragwanath Academic Hospital (CHBAH), Soweto, South Africa [12]. Women included in the study were black South African, living in Soweto, aged 18 years or over, with a spontaneous conception of a singleton pregnancy. Women with a multiple pregnancy, a body mass index >35 kg/m^2^ or an intellectual or physical disability, were excluded. All women had a dating ultrasound scan in the first trimester and HIV testing was routinely offered to those not known to be HIV positive at enrolment. Medical, obstetric and HIV/ART history were collected from medical records, antenatal cards and/or interviews. Perinatal outcomes of interest were recorded at delivery and birthweight of live newborns measured within 12 hours of birth, as previously reported [12].

### Outcome definitions

Preterm birth (PTB) was defined as birth at 16^+0^–36^+6^ weeks’ gestation. Women with moderate-to-late PTB (32^+0^–36^+6^ weeks), very PTB (28^+0^–31^+6^ weeks) or extreme PTB (<28 weeks) were also analysed separately.

### Antiretroviral therapy initiation definitions

HIV positive women were defined as having initiated antiretroviral therapy (ART) preconception (PC) if ART was initiated before the date of the last menstrual period, and antenatal (AN) if ART was initiated after the last menstrual period date.

### Sample collection and processing

Between 27 November 2013 and 20 October 2015, trained study nurses collected peripheral blood samples in each trimester from all women, and at delivery and six weeks postnatally for a subset of HIV positive women. Samples were separated into plasma and peripheral blood mononuclear cells (PBMCs) by standard density gradient centrifugation. PBMCs were frozen in a solution of 50% (v/v) FCS and 10% (v/v) DMSO in R10 media. PBMCs were initially stored at -80°C in South Africa after which samples were shipped to the UK on dry ice where they were stored in liquid nitrogen.

### Flow cytometry

Patient samples were chosen for analysis according to pregnancy outcome, rather than to be representative of enrolled HIV positive or HIV negative women. Frozen PBMCs were thawed in a water bath at 37°C. Each vial of thawed cells (~2.5 x 10^6^ cells) was added to 50 μl of DNAse I solution (1 mg/ml) and resuspended in warm R10 media (37°C). To identify live cells, cells were stained with the Zombie Aqua Fixable Viability Kit [BioLegend] according to the manufacturer's protocol. To identify γδ T cell populations, cells were stained with antibodies specific for CD3 (REA613), TCR γδ (B1), TCR Vδ1 (REA173) and TCR Vδ2 (B6). In addition, cells were stained for CD196/CCR6 (G034E3) [antibodies from BioLegend, except for CD3 and TCR Vδ1 from Miltenyi Biotec]. Cells were incubated with the antibody cocktail in the dark at room temperature for 15 minutes, then washed once in ice-cold staining buffer (PBS with 10% FBS) and fixed in 200 μl of 2% paraformaldehyde. Fixed cells were resuspended in 300 μl ice-cold staining buffer prior to acquisition on a LSR II flow cytometer [Becton Dickinson]. Fluorescence compensation was set using OneComp eBeads™ [Thermo Fisher Scientific], MACS Comp Bead Kit, anti-REA [Miltenyi Biotec], and a single stain of PBMCs using Zombie Aqua™ Fixable Viability Kit [BioLegend]. Flow cytometric analysis, including compensation for spectral overlap, was done using FlowJo V10 software [FlowJo LLC].

### Statistical analysis

Patient characteristics were analysed for normality and compared using the appropriate statistical tests (Mann-Whitney-U test or unpaired t test for continuous variables; Fisher’s exact test or Chi squared test for categorical variables). The Kruskal-Wallis test was used to compare median values of more than two unpaired groups followed by the Dunn’s test for multiple comparisons. P-values <0.05 were considered statistically significant. All statistical analyses were conducted in Graphpad Prism version 8.3.0.

### Ethical approval

Written informed consent was obtained from all women who participated in the study upon enrolment. Ethical approval was obtained from the University of Oxford Tropical Research Ethics Committee (OxTREC) and the Human Research Ethics Committee (Medical) of the University of Witwatersrand, Johannesburg, South Africa.

## Results

### Patient characteristics

A total of 47 HIV positive (HIV+) and 45 HIV negative (HIV-) women were included in this study and characteristics of HIV+ and HIV- women were largely comparable **(Table 1).** The maternal age of HIV+ women was higher than that of HIV- women (p = 0.036) and there was a difference in the years of maternal education (p = 0.006). However, there were no other significant differences, either in obstetric history, number of previous pregnancies, pre-pregnancy BMI, smoking status or alcohol intake **(Table 1)**.

**Table 1:** Patient characteristics.

	HIV+ patients	HIV- patients	Statistical comparison
**Number of patients**	47	45	
**Maternal age** (median [IQR])	33 [28–37]	29 [26–33]	p = 0.036
**Pre-pregnancy body mass index** (mean [SD])	27.7 [4.3]	26.2 [3.5]	p = 0.084
**Number of previous pregnancies** (median [IQR])	2 [1–3]	2 [1–3]	p = 0.902
**History of adverse pregnancy outcome** (number [%])	26 [55%]	28 [62%]	p = 0.402
**Smoking during pregnancy** (number [%])			
Yes	5 [11%]	2 [4%]	p = 0.435
No	42 [89%]	43 [96%]	
**Alcohol intake during pregnancy** (number [%])			
Yes	8 [17%]	4 [9%]	p = 0.355
No	39 [83%]	41 [91%]	
**Number of years of education** (median [IQR])	12 [11–12]	12 [12–12]	p = 0.006
**Antiretroviral therapy initiation category** (number [%])			
Preconception	17 [36%]	N/A	
Post-conception	19 [40%]	N/A	
Unknown	11 [23%]	N/A	
**Number of samples**			
Trimester 1	25	25	
Trimester 2	37	32	
Trimester 3	14	16	
**Weeks + days of gestation at sample collection** (median [range])			
Trimester 1	12+4 [8+0–13+6]	12+0 [8+0–14+2]	p = 0.573
Trimester 2	26+2 [23+4–29+0]	26+0 [20+6–27+6]	p = 0.327
Trimester 3	35+3 [31+1–37+4]	35+4 [30+2–39+0]	p = 0.997
**Preterm birth (PTB)** (number [%])	23 [49%]	25 [56%]	p = 0.525
Moderate-to-late PTB	14 [30%]	10 [22%]	p = 0.409
Very PTB	3 [6%]	6 [13%]	p = 0.311
Extreme PTB	6 [13%]	9 [20%]	p = 0.348

Characteristics of HIV positive and HIV negative pregnant women were compared using the appropriate statistical tests (Mann-Whitney-U test or unpaired t test for continuous variables; Fisher’s exact test or Chi squared test for categorical variables). History of adverse pregnancy outcome: at least one occurrence of preterm birth, low birth weight, miscarriage, stillbirth or neonatal death.

### γδ T cells are stable throughout pregnancy

**Fig 1:**
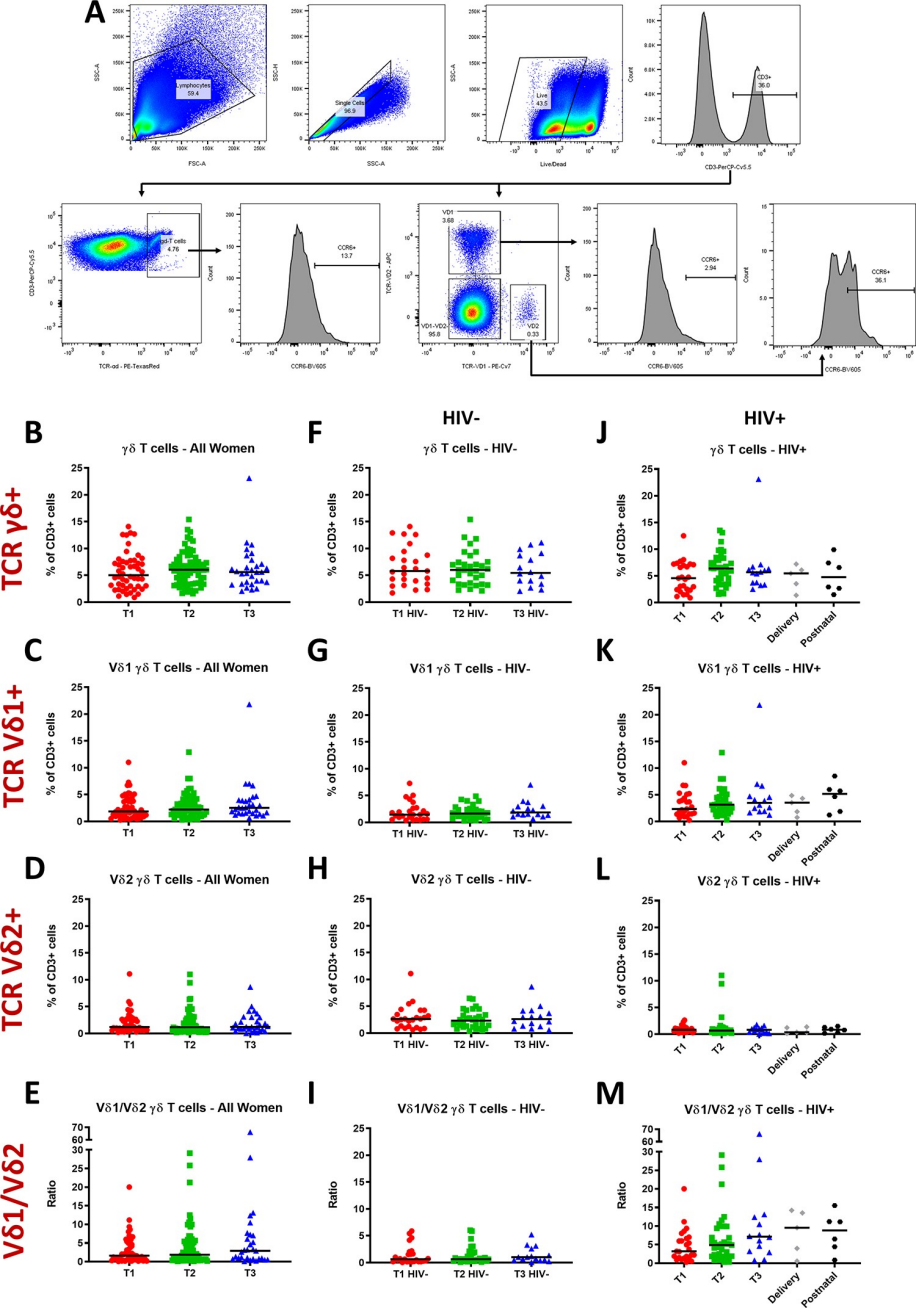
γδ T cells, Vδ1 and Vδ2 γδ T cell subsets and Vδ1/Vδ2 ratios throughout pregnancy. (A) Gating strategy identifying γδ T cells, Vδ1 and Vδ2 γδ T cell subsets, and their CCR6+ subsets, among live CD3+ lymphocytes. (B, F, J) γδ, (C, G, K) Vδ1, and (D, H, L) Vδ2 T cell frequencies as percentages of CD3+ lymphocytes and (E, I, M) Vδ1/Vδ2 ratios, in all women, HIV positive women and HIV negative women, respectively, during the first (T1), second (T2) and third (T3) trimester of pregnancy, and at delivery and six weeks postnatal for HIV positive women. Horizontal bars represent the median. A Kruskal-Wallis test was used to compare values across a trimester, followed by the Mann-Whitney-U test for comparisons between two trimesters. The Mann-Whitney-U test was used to compare delivery or postnatal values with each trimester.

**Fig 2:**
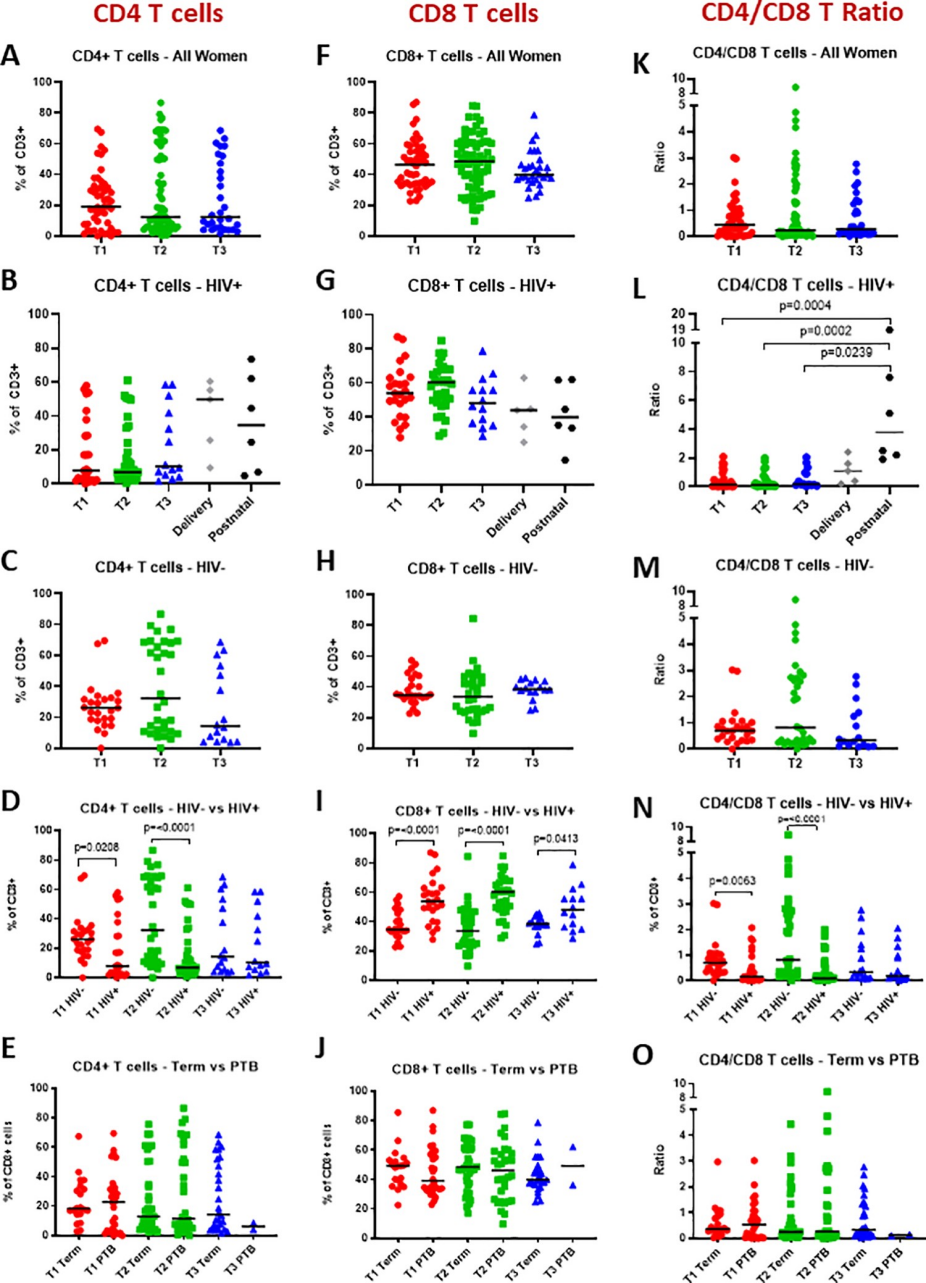
CD4+ T cells, CD8+ T cells and CD4/CD8 ratio throughout pregnancy. (A-E) CD4+, (F-J) CD8+ T cells (% of CD3+ lymphocytes) and (K-O) CD4/CD8 ratio of all women, HIV+ women, HIV- women, HIV+ versus HIV- women and women who delivered at term versus women who delivered preterm, respectively; during the first (T1), second (T2) and third (T3) trimester of pregnancy, and at delivery and six weeks postnatal for HIV positive women.

Total γδ T cells as well as Vδ1 and Vδ2 γδ T cell subsets were identified among CD3+ lymphocytes by flow cytometry (Fig 1A). To begin, global γδ T cells, Vδ1 and Vδ2 cell frequencies and Vδ1/Vδ2 ratios for all women in the study were assessed across pregnancy trimesters 1, 2 and 3. We found that total γδ T cells and Vδ1 and Vδ2 frequencies (expressed as a percentage of CD3+ lymphocytes) and Vδ1/Vδ2 ratios were stable throughout pregnancy with no significant differences in cell frequencies across trimesters (Fig 1B–1E). Similarly, when analysed according to HIV status, cell frequencies were stable throughout pregnancy in both HIV- and HIV+ women (Fig 1F–1M). In HIV+ women, for some of whom delivery and six weeks postnatal samples were available, we also compared pregnancy and non-pregnancy cell frequencies and found that they were comparable (Fig 1J–1M). In addition to γδ T cells, we analysed CD4+ and CD8+ T cell frequencies and the CD4/CD8 ratio in all women and in HIV- and HIV+ women separately. In all groups these cell frequencies and ratios remained stable throughout pregnancy. Six weeks postnatal the CD4/CD8 ratio in HIV+ women was elevated compared to during pregnancy (Fig 2A–2C, 2F–2H and 2K–2M).

### Vδ1 and Vδ2 γδ T cell frequencies are altered in HIV positive pregnant women

**Fig 3:**
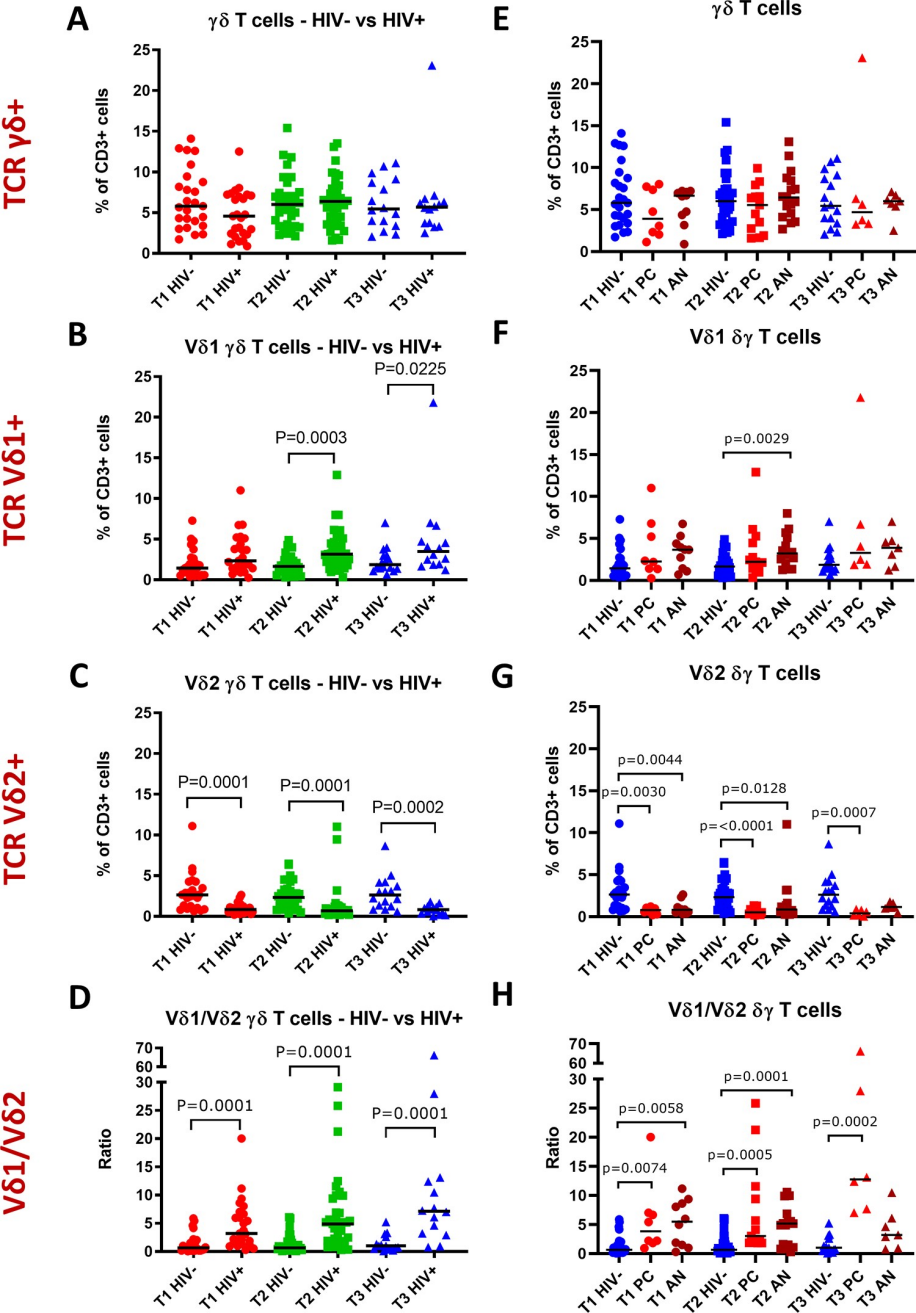
γδ T cells, Vδ1 and Vδ2 γδ T cell subsets and Vδ1/Vδ2 ratios of HIV positive and HIV negative women throughout pregnancy. (A) γδ, (B) Vδ1 and (C) Vδ2 T cell frequencies as percentages of CD3+ lymphocytes and (D) Vδ1/Vδ2 ratios of HIV positive (HIV+) versus HIV negative (HIV-) women during the first (T1), second (T2) and third (T3) trimester of pregnancy. (E) γδ, (F) Vδ1 and (G) Vδ2 T cell frequencies as percentages of CD3+ lymphocytes and (H) Vδ1/Vδ2 ratios during the first, second and third trimester of HIV negative women compared to HIV positive women receiving preconception (PC) or antenatal (AN) antiretroviral therapy. Comparisons between two groups were made using the Mann-Whitney-U test and p-values of significant results are indicated on the graphs.

To investigate the impact of HIV status on γδ T cell frequencies during pregnancy, we next compared total γδ T cells, Vδ1 and Vδ2 cell frequencies as well as Vδ1/Vδ2 ratios at each trimester between HIV- and HIV+ women. While total γδ T cell frequencies were comparable between HIV+ and HIV- women, Vδ1 T cell frequencies were significantly elevated in HIV+ women in the second and third trimester (Fig 3A and 3B), while Vδ2 T cell frequencies were significantly reduced in HIV+ women in all three trimesters (Fig 3C). Correspondingly, there was a highly significant reversal of the Vδ1/Vδ2 ratio in HIV+ women compared to HIV- women across all three trimesters. Specifically, the Vδ1/Vδ2 ratios in HIV+ women in trimesters 1, 2, and 3 were 3.2, 4.9, and 7.1, respectively, compared to 0.6, 0.6 and 1 in HIV- women (Fig 3D). Analysis of CD4+ and CD8+ T cell frequencies demonstrated that CD4+ T cell frequencies were lower in HIV+ women, in the first and second trimester, whereas CD8+ T cell frequencies were higher in HIV+ women across all trimesters (Fig 2D and 2I). Correspondingly, the CD4/CD8 ratio was significantly lower in HIV+ women in the first and second trimester (Fig 2N). Although HIV+ women were older than HIV- women, there was no significant association between maternal age and γδ T cell frequencies in our data set (data not shown).

### γδ T cell frequencies in HIV positive pregnant women are not altered by the timing of antiretroviral therapy initiation

Some reports suggest that the timing of ART initiation, i.e. whether preconception or antenatal, influences risk of adverse pregnancy outcome and in particular preterm birth [6]. Therefore, we compared γδ T cell frequencies of HIV+ women who initiated ART preconception and antenatally. We found that preconception ART initiation did not result in a significant difference in total γδ T cells, Vδ1 and Vδ2 frequencies or Vδ1/Vδ2 ratios when compared to antenatal ART initiation (Fig 3E–3H). However, Vδ1 frequencies were elevated and Vδ2 frequencies reduced in HIV+ women who initiated ART either preconception or antenatally, compared to HIV- women (Fig 3F and 3G). In addition, there was a corresponding reversal in Vδ1/Vδ2 ratio at each trimester in HIV+ women initiating ART either preconception or antenatally compared to HIV- women (Fig 3H).

### γδ T cells are reduced in early pregnancy in women who deliver preterm compared to term

**Fig 4:**
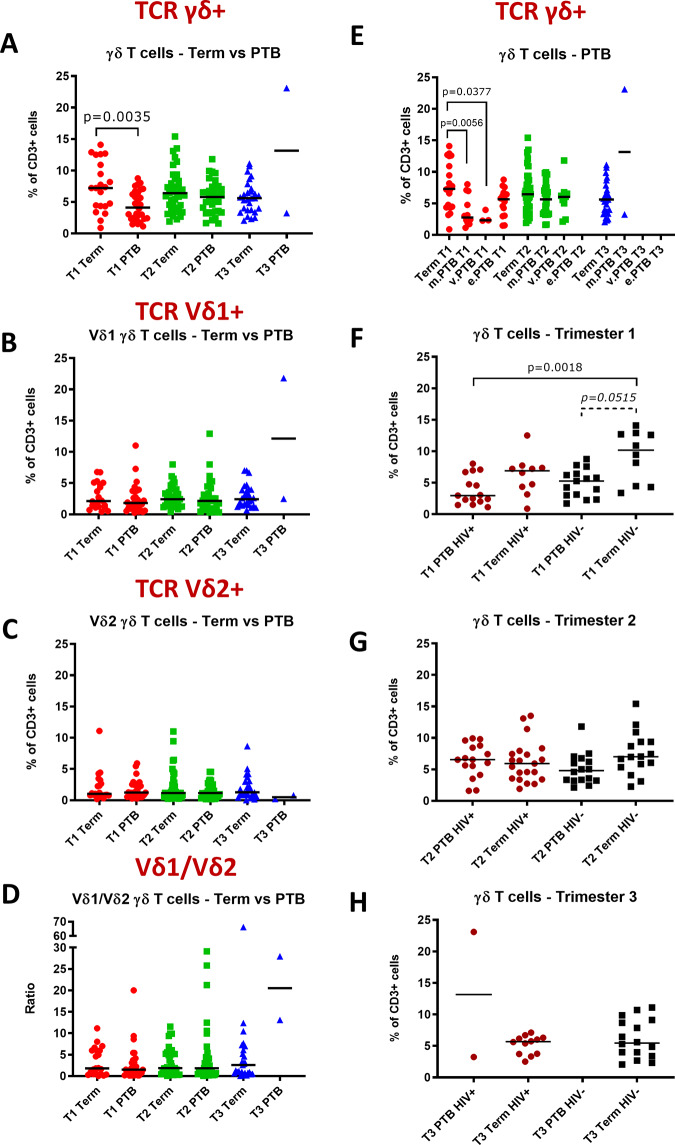
γδ T cells in women with term and preterm births throughout pregnancy. (A) γδ, (B) Vδ1 and (C) Vδ2 T cell frequencies as percentages of CD3+ lymphocytes and (D) Vδ1/Vδ2 ratios of women who delivered at term compared to those who delivered preterm (PTB). (E) γδ T cell frequencies as percentages of CD3+ lymphocytes during the first (T1), second (T2) and third (T3) trimester of women who delivered at term compared to those who had moderately preterm (m.PTB), very preterm (v.PTB), or extreme preterm (e.PTB) births. (F) First, (G) second and (H) third trimester γδ T cell frequencies as percentages of CD3+ lymphocytes of HIV positive (HIV+) and HIV negative (HIV-) women who delivered preterm (PTB) or at term. Comparisons between two groups were made using the Mann-Whitney-U test and p-values of significant results are indicated on the graphs.

As preterm birth is the leading cause of neonatal mortality, with an ill-defined aetiology, and γδ T cells have been implicated in adverse pregnancy outcomes [49, 52], we investigated γδ T cell frequencies in women who delivered preterm. Although Vδ1 and Vδ2 frequencies were comparable between women who delivered preterm and at term, total γδ T cell frequencies were lower in the first trimester in women who delivered preterm compared to term (Fig 4A–4D). Analysis of CD4+ and CD8+ T cell frequencies revealed no significant differences in CD4+ and CD8+ T cell levels or CD4/CD8 ratio between women who delivered preterm and at term (Fig 2E, 2J and 2O).

Total γδ T cell frequencies were then analysed according to the severity of PTB. Women who delivered moderately (32–36 weeks) and very (28–31 weeks) preterm had significantly lower total γδ T cell frequencies in the first trimester than women who delivered at term (Fig 4E). When analysed according to HIV status, HIV- women who delivered preterm showed a trend for lower γδ T cell frequencies than HIV- women who delivered at term and this was particularly evident in the first trimester (p = 0.0515) (Fig 4F and 4G). γδ T cell frequencies were also lower in the first trimester in HIV+ women who delivered preterm compared to HIV+ women who delivered at term, but this difference was not statistically significant (Fig 4F). However, first trimester total γδ T cell frequencies were lowest in HIV+ women who delivered preterm, which was significantly lower than γδ T cell frequencies in HIV- women who delivered at term (p = 0.0018) (Fig 4F). In the third trimester there were too few PTB data points to draw any conclusions, due to delivery before planned third trimester sample collection (Fig 4H).

### CCR6+ Vδ1 γδ T cells are reduced in HIV positive women and women who deliver preterm

**Fig 5:**
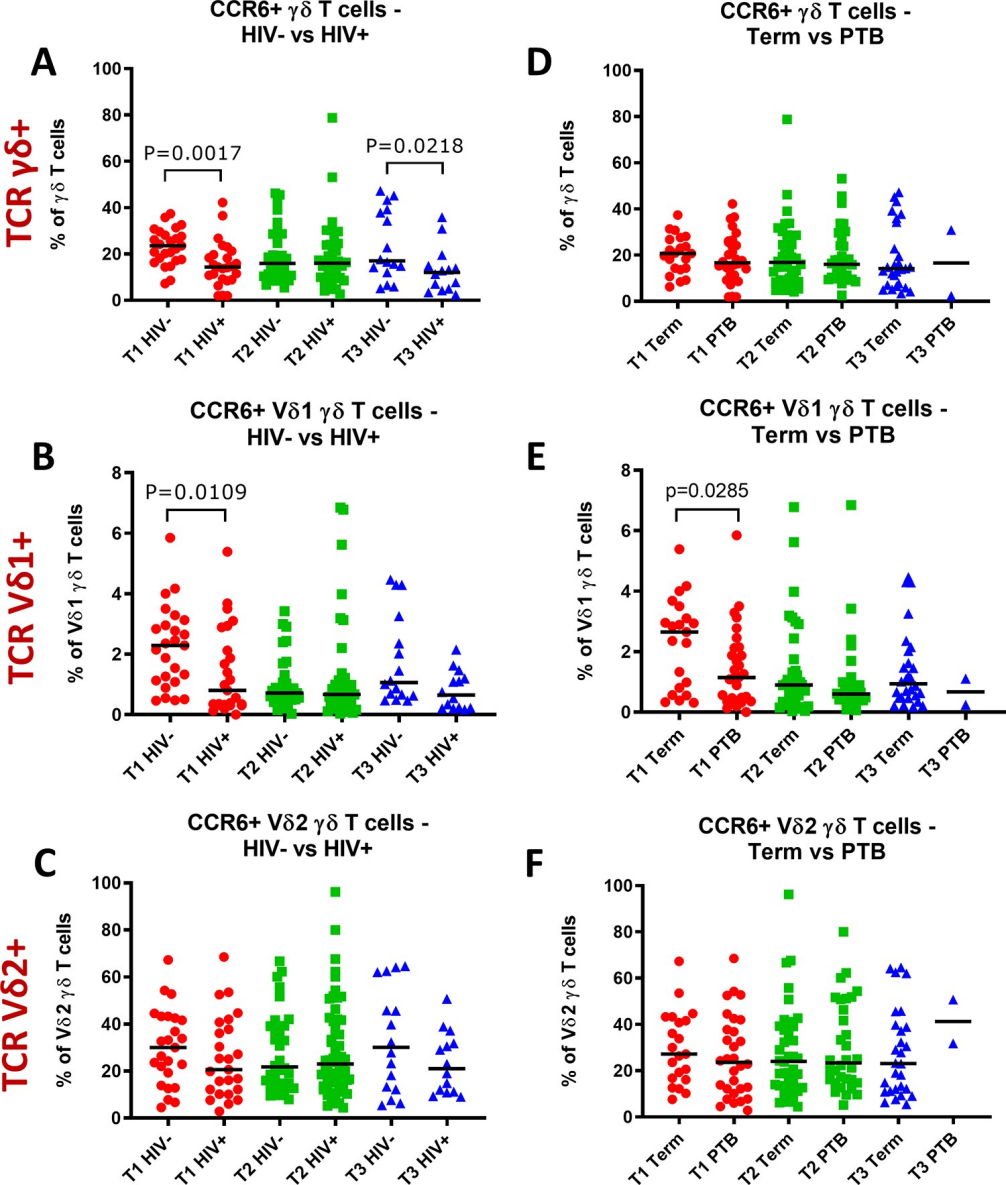
CCR6+ γδ T cells of HIV positive and HIV negative women or women with term or preterm births throughout pregnancy. CCR6+ (A, D) γδ, (B, E) Vδ1 and (C, F) Vδ2 T cell frequencies as percentages of total γδ, Vδ1, and Vδ2 γδ T cells respectively, of HIV positive (HIV+) versus HIV negative (HIV-) women (A-C) or women who delivered at term compared to those who delivered preterm (PTB) (D-F), during the first (T1), second (T2) and third (T3) trimester. Comparisons between two groups were made using the Mann-Whitney-U test and p-values of significant results are indicated on the graphs.

γδ T cells can express the chemokine receptor CCR6 [53, 54], which is involved in lymphocyte trafficking, therefore, we hypothesised its expression on γδ T cells may be relevant to preterm birth in the potential recruitment of γδ T cells from the periphery to sites such as the maternal-fetal interface. The proportion of total γδ T cells that were CCR6+ was significantly lower in the first and third trimesters in HIV+ women compared to HIV- women (Fig 5A). The proportion of Vδ1+ T cells that were CCR6+ in the first trimester was also significantly lower in HIV+ women compared to HIV- women (Fig 5B), but this was not statistically significant for Vδ2+ T cells (Fig 5C).

Moreover, the proportion of Vδ1+ T cells that were CCR6+ was significantly lower in the first trimester in women who gave birth preterm compared to term (Fig 5E). In contrast, the proportion of CCR6+ cells among total γδ T cells and Vδ2+ T cells were similar for women who delivered preterm and term throughout pregnancy (Fig 5D and 5F).

**Fig 6:**
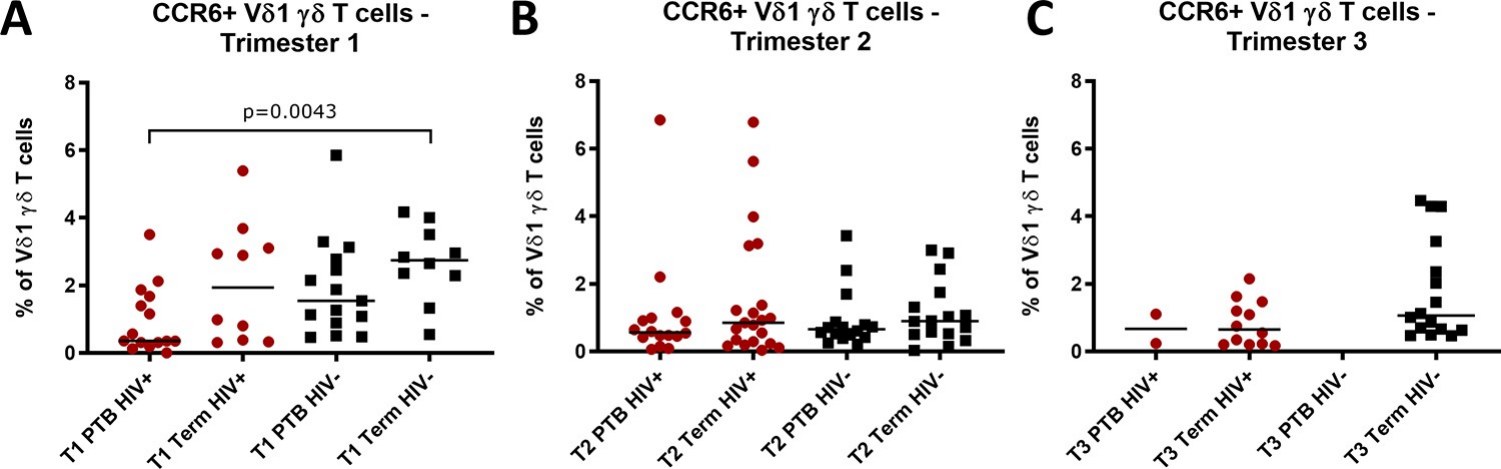
CCR6+ Vδ1 γδ T cells of HIV positive and HIV negative women who delivered preterm or at term. (A) First (T1), (B) second (T2) and (C) third (T3) trimester CCR6+ Vδ1 γδ T cell frequencies as a percentage of Vδ1 γδ T cells, of HIV positive (HIV+) and HIV negative (HIV-) women who delivered preterm (PTB) or at term. Comparisons between two groups were made using the Mann-Whitney-U test and p-values of significant results indicated on the graphs. P-values in italics with dashed brackets indicate differences nearing statistical significance.

Given the association of the proportion of Vδ1+ T cells that were CCR6+ with HIV infection and PTB, we also analysed CCR6+ Vδ1+ T cells according to combined HIV and preterm status. In the first trimester, the proportion of Vδ1+ T cells that were CCR6+ was lowest in HIV+ women who delivered preterm and highest in HIV- women who delivered at term (p = 0.0043) with intermediate levels for HIV+ women who delivered at term and HIV- women who delivered preterm (Fig 6A). No differences were seen in the second and third trimesters (Fig 6B and 6C).

## Discussion

To our knowledge this is the first report investigating the relationship between γδ T cell frequencies, HIV infection and preterm birth, using blood samples collected throughout the course of pregnancy. A previous report has shown that total γδ T cells are unchanged at delivery between pregnant HIV negative and HIV positive women [55] and we have taken this further by investigating total γδ T cell frequencies, as well as Vδ1 and Vδ2 frequencies, Vδ1/Vδ2 ratios and the CCR6 marker, at each trimester and at delivery and six weeks postnatal for a subset of HIV positive women.

We found that total γδ T cell levels were similar between HIV positive and HIV negative women. However, throughout pregnancy maternal HIV infection was associated with reduced levels of the Vδ2+ subset and increased levels of the Vδ1+ subset, leading to a reversal of the Vδ1/Vδ2 ratio. Timing of ART initiation among HIV positive women did not affect levels of γδ T cells, the Vδ1+ and Vδ2+ subsets, or the Vδ1/Vδ2 ratio. Importantly, we report for the first time that preterm birth is associated with lower total γδ T cell levels in early pregnancy and that γδ T cell frequencies are lowest in HIV positive women who deliver preterm. Moreover, in the first trimester the proportion of Vδ1+ T cells that were CCR6+ was reduced in HIV positive women and women who delivered preterm, resulting in the lowest proportion of CCR6+ Vδ1+ T cells in HIV positive women who delivered preterm. Our findings suggest that altered γδ T cell frequencies may link maternal HIV infection and preterm birth, and γδ T cell frequencies in early pregnancy may serve as predictive biomarkers for women at risk of delivering preterm.

The underlying disease mechanisms of preterm birth and the role of γδ T cells in pregnancy remain ill defined, however, peripheral γδ T cell frequencies have been shown to increase in pregnancy compared to non-pregnant women [49, 56, 57]. While some studies do not report a pregnancy associated increase in γδ T cells [58], it may be expected that a reduction in γδ T cells might be associated with pathology. And indeed we demonstrate that lower total γδ T cell frequencies in the first trimester occur in women who experience preterm birth, predominantly moderately (32–36 weeks) and very (28–31 weeks) preterm birth. When stratified according to both HIV and preterm birth status, the lowest total γδ T cell frequencies were found in HIV positive women who delivered preterm, jointly followed by HIV positive women who delivered at term and HIV negative women who delivered preterm. The highest γδ frequencies were found in HIV negative women who delivered at term. This stepwise decrease in γδ T cells with increasing pathology (i.e. HIV infection and preterm birth), such that the lowest frequencies occur in women who experience preterm birth in combination with HIV infection, suggests a link between the HIV-induced mechanisms that decrease total γδ T cells in early pregnancy and the mechanisms that contribute to preterm birth. Other reports also suggest decreases in γδ T cell frequencies are associated with adverse pregnancy outcomes. For instance, Talukdar et al. found that pregnant women with a history of recurrent pregnancy loss had a significant reduction in peripheral blood total γδ T cell frequencies [49].

In non-pregnant women Vδ2 γδ T cells predominate in the peripheral blood but in healthy pregnant women Vδ1+ cells form the major γδ T cell population [51]. A continued dominance of peripheral Vδ2+ subsets in pregnancy is associated with recurrent pregnancy loss; therefore, a Vδ1 bias is thought to be required for the maintenance of a successful pregnancy [56, 57]. In the decidua, γδ T cells are a source of IL-10 which promotes trophoblast proliferation and invasion and suppresses trophoblast apoptosis [59]. Increases in peripheral γδ T cells may originate from the decidua in which Vδ1 T cells also predominate [51, 60], therefore, the presence and role of γδ T cells in the periphery may mirror that of γδ T cells in the decidua. This would suggest peripheral γδ T cell frequencies may not only be indicative of pathology such as preterm birth but be reflective of processes at the maternal-fetal interface at a site that is easier to access. Recognition of HLA-E on trophoblasts via the inhibitory receptor CD94/NKG2A expressed on γδ T cells is proposed to result in the activation of Vγ1Vδ1 T cells leading to increased IL-10 production and a decrease in cytotoxic function. In contrast, HLA-E recognition by Vγ9Vδ2 T cells suppresses their cytotoxic and Th1 cytokine response, thereby promoting a favourable immune environment for the maintenance of a successful pregnancy [56, 57]. Vδ2 T cells from women who suffer recurrent pregnancy loss have been shown not to recognise HLA-E potentially resulting in a lack of inhibition of cytotoxicity and a role in inadequate immune-protective responses to the conceptus [61]. In our cohort, Vδ1 frequencies were greater than Vδ2 in HIV positive women but similar among women who delivered preterm and at term. However, the cytotoxic potential of the Vδ2 γδ T cells in women who delivered preterm is unknown. This is an area for further investigation to elucidate the mechanisms of preterm birth.

Despite a significant reduction in first trimester total γδ T cell frequencies in women who delivered preterm, corresponding Vδ1 and Vδ2 frequencies were comparable between women who delivered at term and preterm. While γδ T cells are commonly categorised as Vδ1 or Vδ2 according to their TCR δ chain usage, in humans Vδ3 is another commonly used δ chain alongside the less frequently used Vδ4, Vδ5, Vδ6, Vδ7 and Vδ8 chains [62]. Therefore, the change in total γδ T cell frequencies could be composed of changes in the frequency of alternative Vδ chains and is a relevant area for further investigation.

In addition to first, second and third trimester samples, we also had delivery and six week postnatal samples for a limited number of HIV positive women. This allowed us to track γδ T cell frequencies throughout pregnancy and into the post-partum period. All γδ T cell frequencies remained stable across pregnancy as well as between pregnancy and delivery or postnatal periods. Other groups have reported a pregnancy-associated increase in peripheral γδ T cells and increased postpartum Vδ1 frequencies compared to the third trimester and non-pregnant women [49, 50]. Although we did not observe similar changes, this could have been down to the low number of samples in our delivery and postnatal groups. In addition, our study made comparisons between pregnancy and a period outside of pregnancy in a subset of our patients, rather than to a non-pregnant control group. Also the women in the studies mentioned were healthy, therefore, a lack of postpartum increase in γδ T cells, as reported by Tham et al. [50], could be a feature of HIV infection.

Some reports have suggested a potential link between adverse pregnancy outcomes and the timing of ART initiation, i.e. preconception or antenatal [6]. At each trimester we found that the γδ T cell frequencies assessed were comparable between HIV positive women who initiated ART preconception and antenatally. Therefore any differences in adverse pregnancy outcomes that may relate to the timing of ART initiation do not appear to involve changes in γδ T cell frequencies.

CCR6, expressed on T cell subsets including γδ T cells, is a chemokine receptor for CCL20 and in humans appears to be involved in the recruitment of immune cells to sites of injury [63, 64]. Unlike total γδ T cell frequencies, which were unchanged between HIV positive and HIV negative women, the proportion of γδ T cells which were CCR6+ were significantly reduced in HIV positive women in the first as well as the third trimester. In the first trimester this was largely due to significant decreases in the proportion of CCR6+ Vδ1+ cells, which were similarly reduced in women who delivered preterm compared to term. Furthermore, when stratified according to combined HIV and preterm birth status, first trimester CCR6+ Vδ1 T cells were lowest in HIV positive women who delivered preterm. Therefore, our data indicate that reduced CCR6+ Vδ1+ frequencies in early pregnancy may be a link between maternal HIV infection and preterm birth. Similarly, CCR6 mRNA expression has been shown to be reduced in the peripheral blood and decidua of women who experience unexplained recurrent miscarriage compared to women with healthy pregnancies [65].

During thymic development in murine models, γδ T cells differentiate into antigen-naïve IL-17 producing cells or antigen-experienced IFN-γ producing cells [66]. In these γδ T cells, IL-17 production is associated with the surface expression of CCR6, while IFN-γ production is associated with NK1.1 expression [67]. While investigations into murine CCR6+ γδ T cells may not directly correlate with human γδ T cells, in healthy human donors a proportion of Vγ9Vδ2 T cells stain positively for intracellular IL-17 upon ex vivo PMA/ionomycin stimulation [68]. Furthermore, cytoplasmic expression of IL-17 has also been demonstrated in Vδ1 T cells of HIV positive individuals [54]. Placental angiogenesis is a process that is vital to the healthy development of the placenta and therefore the maintenance of a successful pregnancy. IL-17 has been shown to induce neovascularisation and the production of pro-angiogenic molecules [69]. At the maternal-fetal interface decidual stromal cells recruit IL-17-producing Th17 cells from the periphery into the decidua where they promote the survival, growth and function of trophoblast cells via IL-17 during the first trimester of pregnancy [46]. γδ T cells are also present within the decidua [16] and a decrease in their number may be detrimental to processes which support the maintenance of healthy pregnancies. Such as a decrease in CCR6+ Vδ1 γδ T cells with the potential to produce IL-17 and which we find are reduced in the first trimester of both HIV positive women and women who deliver preterm. Further investigations are required into the connection between IL-17-producing γδ T cells, HIV infection and pregnancy outcome.

In summary, our data indicate that a low frequency of γδ T cells and a low proportion of CCR6+ Vδ1+ cells in the first trimester of pregnancy are associated with maternal HIV infection and preterm birth. Importantly, we demonstrate that the associations of γδ T cells and CCR6+ Vδ1 T cells with preterm birth are not related to HIV disease progression, as indicated by CD4 counts and CD4/CD8 T cell ratios, but rather they are specific to preterm birth. The precise mechanisms of how changes in γδ T cells may contribute to adverse pregnancy outcomes are currently unknown. Functional studies, including investigations into the cytokine production of γδ T cells and CCR6+ Vδ1+ cells, according to preterm birth and HIV status, are required to help further understand the complex role of γδ T cells in HIV infection and preterm birth. In addition, γδ T cell frequencies in early pregnancy may serve as predictive biomarkers to identify women at risk of delivering preterm.
